# Accuracy of training recommendations based on a treadmill multistage incremental exercise test

**DOI:** 10.1371/journal.pone.0204696

**Published:** 2018-10-11

**Authors:** Hendrik Mugele, Ashley Plummer, Omar Baritello, Maggie Towe, Pia Brecht, Frank Mayer

**Affiliations:** 1 Department of Sport and Health Sciences, Clinical Exercise Science, University of Potsdam, Potsdam, Germany; 2 Department of Sport and Health Sciences, Professorship of Sports Medicine and Orthopedics, University Outpatient Clinic, University of Potsdam, Potsdam, Germany; Nottingham Trent University, UNITED KINGDOM

## Abstract

Competitive runners will occasionally undergo exercise in a laboratory setting to obtain predictive and prescriptive information regarding their performance. The present research aimed to assess whether the physiological demands of lab-based treadmill running (TM) can simulate that of over-ground (OG) running using a commonly used protocol. Fifteen healthy volunteers with a weekly mileage of ≥ 20 km over the past 6 months and treadmill experience participated in this cross-sectional study. Two stepwise incremental tests until volitional exhaustion was performed in a fixed order within one week in an Outpatient Clinic research laboratory and outdoor athletic track. Running velocity (IAT_speed_), heart rate (IAT_HR_) and lactate concentration at the individual anaerobic threshold (IAT_bLa_) were primary endpoints. Additionally, distance covered (DIST), maximal heart rate (HR_max_), maximal blood lactate concentration (bLa_max_) and rate of perceived exertion (RPE) at IAT_speed_ were analyzed. IAT_speed_, DIST and HR_max_ were not statistically significantly different between conditions, whereas bLa_max_ and RPE at IAT_speed_ showed statistical significance (p < 0.05). Apart from RPE at IAT_speed_, IAT_speed_, DIST, HR_max_ and bLa_max_ strongly correlate between conditions (r = 0.815–0.988). High reliability between conditions provides strong evidence to suggest that running on a treadmill are physiologically comparable to that of OG and that training recommendations and be made with assurance.

## Introduction

General exercise examinations such as multistage incremental exercise testing (IET) are common practice for assessing recreational and professional athletes [1]. IET can be used to identify current training status, predict performance capability and help to give training recommendations [1,2]. There are several field and laboratory protocols that are currently implemented and consist of many differentiating variables including stage duration, number of stages, speed increments, starting velocities and inclinations [3,4]. In clinical exercise science, the use of motorized treadmills in a laboratory setting is a widely accepted method as it is well standardized, reproducible and facilitates the measurement of targeted performance parameters, e.g. heart rate, ventilation and blood lactate (bLa) [5].

It is of interest for athletes and coaches alike to be able to identify the overall endurance capacity, which has been defined as the highest constant exercise intensity that can be maintained for a sustained duration without a continuous rise in bLa [1]. Lactate related thresholds strongly correlate with endurance performance and therefore directly pertain to training recommendations [1,3,6]. As described by Dickhuth (1999), the individual anaerobic threshold (IAT) is one of many concepts that can estimate the highest intensity at which the lactate equilibrium is maintained [7]. Due to a close relationship between lactate threshold values and maximal lactate steady state (MLSS) [1], defining the running speed at which the IAT is achieved (IAT_speed_) is a useful indicator not only because it provides a quantifiable reference speed to which endurance athletes can optimally train at, but can also serve as a predictor for endurance performance times [8]. With a defined IAT_speed_, athletes of various abilities can train efficiently to continuously improve endurance performance without overreaching or overtraining [9]. Furthermore, to prescribe tolerable and safe exercise interventions in various patient groups assessing bLa can be an useful tool as well [5,10–13].

It must be considered; however, that providing training recommendations based on laboratory IET can only be valid if the conditions accurately simulate that of overground running (OG). There has been some evidence to suggest inherent biomechanical differences between OG and running on a motorized treadmill [14,15]. For instance, OG results in an increased peak hip flexion and flexion angle at footstrike; in turn, this might be attributable to the shorter stride length observed on a treadmill [15]. These factors invariably affect running efficiency and therefore, impact the metabolic cost of running. Furthermore, whilst running at speeds above 10.5 km/h during OG, wind and air resistance can serve as major confounding factor in field tests [16–18]. Applying a treadmill gradient of 1% has been shown to compensate for the reduced metabolic costs due to the missing air and wind resistance in a laboratory setting [2]. However, characteristics of each treadmill depend on the manufacturers specification (i.e. magnitude of belt friction and fluctuations in speeds) so this gradient may not necessarily be universally applicable. Furthermore, long-term treadmill use can result in degradation or "wear and tear" of its components. If differences between treadmills can be reasonably assumed, then it becomes necessary to revalidate the device commonly used in day-to-day practice and, in turn, supporting the validity of training recommendations. Treadmill used in laboratory settings will regularly apply a slight pre-defined gradient to compensate these potential confounding factors. This notion was supported by internal unpublished work obtained in partial fulfilment of the Clinical Exercise Science Program of the University, which determined that a 0.4% gradient was a more suitable incline as opposed to that proposed by Jones and Doust (1996) [2]. However, periodic validation against OG is mandatory in order to assess the accuracy of treadmill-based training recommendations. Specifically, determining whether the gradient of 0.4% over- or underestimates the true endurance performance as expressed by the IAT_speed_.

Therefore, the aim of the present study was to revalidate the frequently used IET on a motorized treadmill that is used during pre-participation sports examination and annual health evaluation as well as performance diagnostics. It was hypothesized that the treadmill protocol could be reproduced successfully on the track. Furthermore, a gradient of 0.4% would account for the air and wind resistance; therefore, no difference between conditions would be observed.

## Methods

### Subjects

A sample size of 12 was determined using a priori two-sided power analysis (G*Power (3.1 software; Düsseldorf, Germany) which would achieve 80% power at the significance level of 0.05. A review of relevant literature revealed a strong relationship between running velocity and the IAT (i.e. r = 0.91) [1]. To account for possible dropouts, a total of 15 participants were recruited for the study. The inclusion criteria were: (i) experienced runner with an average weekly mileage of equal or greater than 20 km of just running [19], (ii) the weekly mileage is being consistently achieved for at least half a year, (iii) prior experience and confidence with treadmill running, (iv) actively engaged in competition/training and (v) no performance inhibiting injury. The study was approved by the ethical committee of the University Potsdam and written informed consent was obtained from all participants.

### Experimental design

Each participant initially performed the IET on a treadmill in the laboratory setting (TM) followed by the same protocol in the field (RT). The order of the trials was fixed and not randomized because weather conditions had been controlled as much as possible. The test began at a running velocity of 6 km/h with a progressively increasing speed of 2 km/h every 3 minutes until volitional termination [8]. Immediately following each stage, there was a 30 second break period to taking bLa measurements and subjective measures of the participants rate of perceived exertion (RPE) via BORG’s RPE-Scale 6–20 [20]. The primary outcome measures of interest were the running velocity, heart rate and bLa concentration at the IAT (IAT_speed_, IAT_HR_ and IAT_bLa_ respectively). The secondary outcome variables were the maximal heart rate (HR_max_), maximal blood lactate (bLa_max_), stages reached in both conditions in meters as well as final running velocity and RPE. The participants were verbally encouraged to give their maximal effort particularly towards the end the end of the protocol when they were visibly waning. All participants were tested within one week (3.2 ± 0.8 days; range 2–5 days), which should have allowed for adequate recovery. Prior to each measurement, a physician performed a physical examination in order to assure that participants can perform maximal exercise by checking for possible orthopedic and cardiopulmonary contraindications for exercise testing.

### Laboratory test

TM was conducted on a motorized treadmill (Pulsar, h/p/cosmos Sports & Medical, Nussdorf-Traunstein, Germany; speed accuracy ±5%) with a constant gradient of 0.4% (mean ambient temperature ± standard deviation (SD): 25 ± 1°C, range 23–26°C). Heart rate (HR) measurements were taken 15 seconds before the end of each stage. BORG readings and capillary blood samples from the earlobe were taken within the 30 seconds break period. Maximum running velocity was linearly interpolated when premature termination occurred by multiplying the time spend at that stage with the quotient of stage duration and speed increment (0.0031 m/s). For instance, if termination occurred at stage 7 (i.e. 18 km/h) after 0:38 min, the maximum running velocity of 18.42 km/h was achieved. Participants had been asked to abstain from exercise the day before the test. Additionally, they had been asked not to deviate from their regular nutritional habits and to be fasted three hours before the test. They were allowed to drink ad libitum. However, caffeinated beverages on the day of the test were not permitted. The hydration status was not controlled.

### Field test

RT was performed within seven days after TM (mean ambient temperature: 20 ± 4°C, range: 16–27°C). Participant’s preparation was the same as in TM. The test itself was performed on a 400m tartan athletic outdoor running track. In order to adjust and maintain athletes’ running speed, an acoustic signal sequence (i.e. audio beeps) was pre-recorded using commercially available music editing software (Garage Band (vers. 10.1.6). Apple Inc., California, United States of America). The audio file was played on a personal mobile phone (IPhone 5s, Apple Inc., California, United States of America) which was connected to a Bluetooth multimedia speaker (JBL charge3+. Harman International Industries, Incorporated. Stamford, United States of America). To ensure further proper speed defaults, a road bike was equipped with the speaker and a calibrated cycling computer (CUBE RACE TRAIN. Pending System GmbH & Co. KG. Waldershof, Germany). The pacing bike stayed in synchronization with the audio beeps and would cycle alongside the athlete giving verbal instruction and encouragement if the participant started to fall behind the target speed. The sequence was designed to sound every 50 m as indicated by a cone. If (via the sound of the audio beep) the participants failed to reach the cone on two consecutive occasions due to fatigue, the test was terminated. The distance from the beginning to the dropout point of the last stage was recorded. At this point, the meters achieved could be converted into time completed at the given stage. Capillary blood samples, HR and BORG readings were taken within 30 seconds of inactivity between stages. If participants did not finish an increment their maximal running velocity was linearly interpolated.

### IAT

The IAT was determined according to the Dickhuth method [7]. Specifically, the authors proposed that the IAT was located 1.5mmol/l above the minimum lactate equivalent. The calculation was determined using Ergonizer Software (Ergonizer, Freiburg, Germany) [21].

### BLa and HR measurements

All measurements of bLa were assessed in a standardized manner in both RT and TM conditions at baseline, after every increment, immediately after termination, three and five minutes post-test. The procedure was as follows (i) the ear was initially sterilized with the use of alcohol wipes and wiped dry with a tissue, (ii) after piercing the skin with a lancet, the first drop of blood was discarded and a 20μl blood sample was collected using a plastic capillary tube end-to-end (>1% sodium heparin; EKF Diagnostic Sales, Magdeburg, Germany), (iii) the capillary tube was then placed in a deproteinizing agent and finally (iv) analysis of the blood lactate was conducted using the enzymatic-amperometric method (Biosen S-line, EKF Diagnostic Sales, Magdeburg, Germany; coefficient of variance: 1.5%).

HR was continually measured in both conditions using a commercially available wrist unit and transmitter (F6, Polar electro, Kempele, Finland; accuracy ±1 beat per minute (bpm)). In both protocols, readings were taken at the end of each stage, three and five minutes after volitional termination.

### RPE

BORG’s RPE-Scale 6–20 [20] was used for each increment in order to get a subjective measure of the participant’s perceived exertion. The participants were required to state the corresponding number at the end of each stage in both testing conditions.

### Statistical analysis

The results for all participants are presented as mean ± SD if not stated otherwise. All data was checked for normal distribution using the Shapiro-Wilk test with a significance level set at p< 0.05. Differences between conditions for each primary and secondary variable were compared using either paired *t* test or a non-parametric Wilcoxon signed rank test.

To quantify the agreement of the outcome measures between TM and RT, a Bland-Altman analysis was conducted. Priori defined 95% limits of agreement (LoA) of ±1km/h for IAT_speed_ were considered to be of relevance [22,23]. The strength of the relationship between the primary outcome measures of the two running conditions was analyzed with the Pearson product-moment correlation. The strength of the correlation was determined as follows: r = 0 (no), r = 0.2 (weak) r = 0.50 (moderate); r = 0.8 (strong) and r = 1 (perfect) [24]. The primary data entry and descriptive calculations were done using Microsoft Excel for Mac 2011 (Version 14.6.1 (160122)). Further statistical analyses were performed using IBM SPSS Statistics for Macintosh (Version 21.0. Armonk, NY: IBM Corp.).

## Results

### Subjects

A total of 15 participants (seven females and eight males) completed the study without adverse events. The baseline characteristics including anthropometric data are shown in Table 1. All participants were recreational athletes, of which, six were from predominantly team sports (i.e. soccer, hockey and volleyball) and nine from individual sports (i.e. running, triathlon, obstacle running, cycling and Cross Fit).

**Table 1:** Baseline characteristics.

		Gender	
	Total *(N = 15)*	Males *(N = 8)*	Females *(N = 7)*
**Age [yrs.]**	30 (27–34)	29 (25–30)	36 (31–38)
**Height [cm]**	180 (± 10)	180 (± 10)	170 (± 10)
**Weight [kg]**	67.7 (± 9.6)	75.0 (± 4.7)	59.2 (± 5.8)
**Body Mass Index [kg/m**^**2**^**]**	22.3 (20.4–22.8)	22.7 (21.3–23.2)	21.1 (19.9–22.3)
**Training volume [h/wk.]**	5 (4–7)	5 (4–6)	5 (4.5–8.5)
**Mileage [km/wk.]**	25 (20–36)	28 (20–38)	20 (20–30)

Age, Body Mass Index, training volume and mileage are presented as median (interquartile range). Height and weight are presented as mean (± standard deviation).

### IAT_speed_

**Fig 1:**
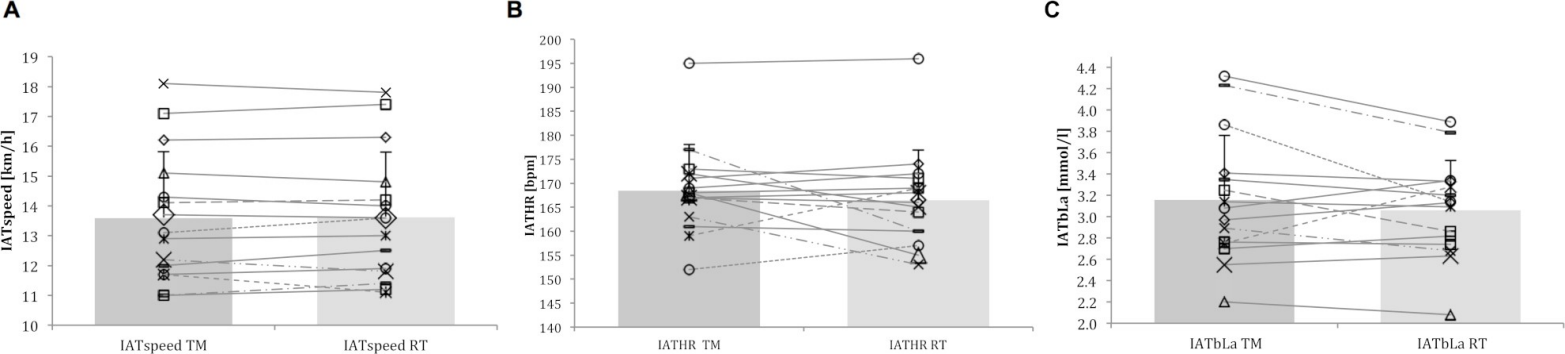
Individual and overall mean performance in TM and RT including standard deviation. (A) IAT_speed_, (B) IAT_HR_ and (C) IAT_bLa_.

**Fig 2:**
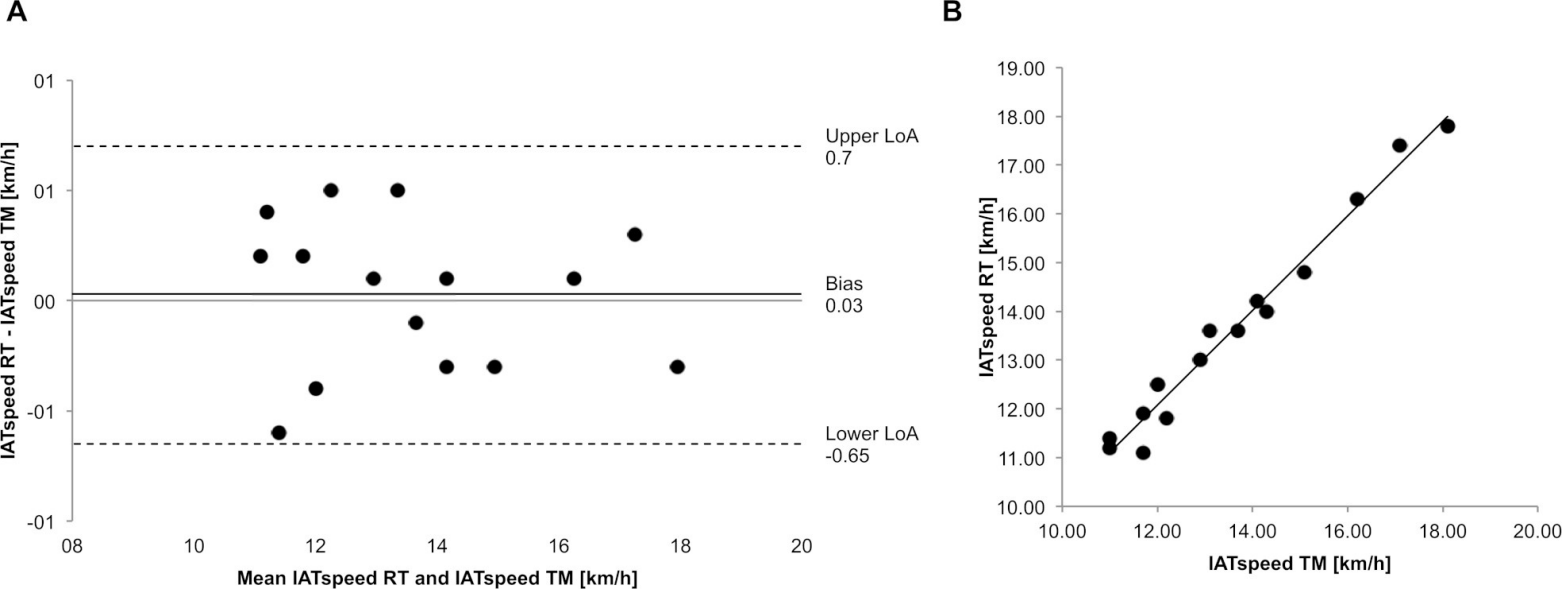
Agreement and strength of the relationship between TM and RT. (A) Bland-Altman plot to show the agreement of IAT_speed_ and (B) Scatterplot of the IAT_speed_ achieved for RT (y-axis) versus TM (x-axis) to show strength of relationship.

The Shapiro-Wilk test showed that data is normally distributed (ΔRT *&* TM: p = 0.389). A two-tailed paired *t* test revealed no statistically significant difference between TM (mean ± SD, 95% confidence interval (CI): 13.61 ± 2.21, 12.39–14.83 km/h) and RT (13.64 ± 2.17, 95% CI 12.43–14.84 km/h; p = 0.768; Fig 1A). Six and nine participants achieved a right shift of their IAT_speed_ in TM and RT, respectively. The Bland-Altman plot (Fig 2A) showed a systematic bias of 0.03 km/h with a random error of 0.67 km/h (upper LoA: 0.7, lower LoA: -0.65 km/h). Correlation analysis (Fig 2B) shown a strong positive association (r = 0.988; p< 0.001) with 97.6% of the variability of IAT_speed_ of the RT condition being explained by the IAT_speed_ achieved in the TM.

### IAT_HR_

**Fig 3:**
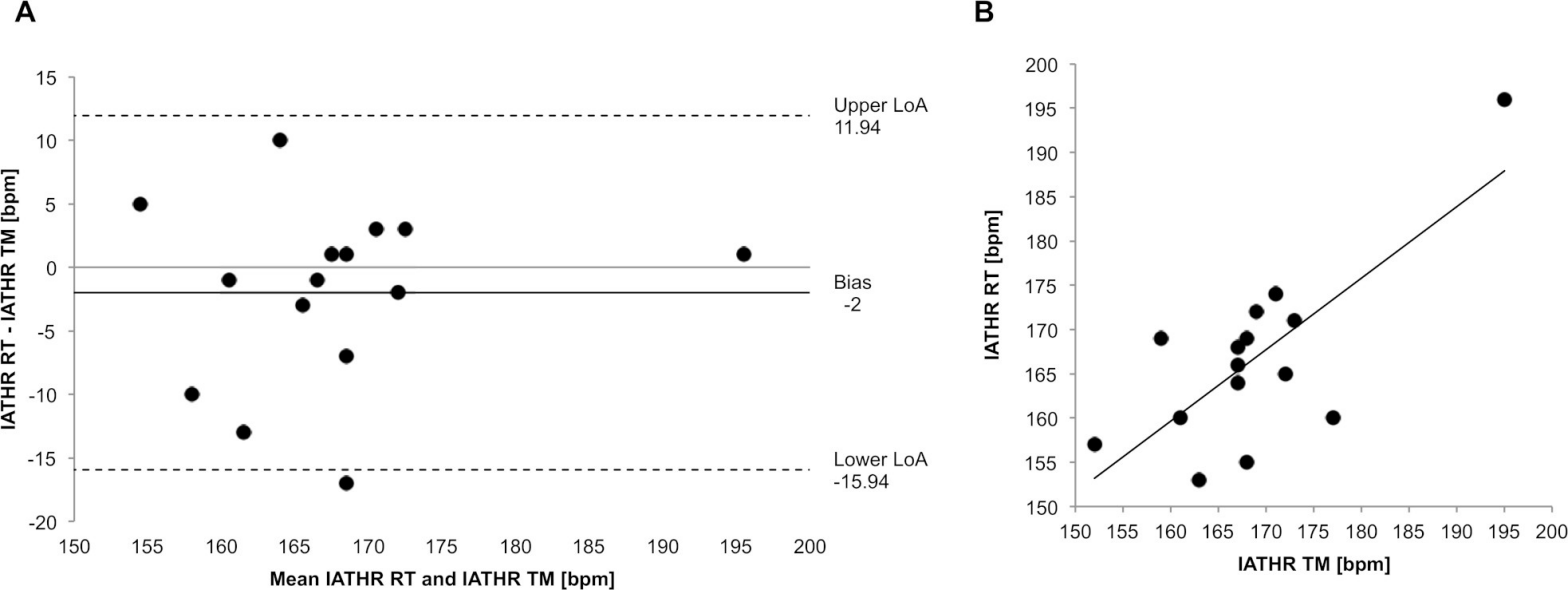
Agreement and strength of the relationship between TM and RT. (A) Bland-Altman plot to show the agreement of IAT_HR_ and (B) Scatterplot of the IAT_HR_ achieved for RT (y-axis) versus TM (x-axis) to show strength of relationship.

IAT_HR_ was normally distributed (ΔRT *&* TM: p = 0.453). The *t* test revealed no statistically significant difference between TM (169 ± 10, 95% CI 163–174 bpm) and RT (167 ± 10, 95% CI 161–172 bpm; p = 0.294; Fig 1B). The Bland-Altman plot (Fig 3A) showed a systematic bias of -2 bpm with a random error of 13.9 bpm (upper LoA: 11.9, lower LoA: -15.9 bpm). Correlation analysis (Fig 3B) shown a moderate association (r = 0.746; p = 0.001) with 55.6% of the variability of IAT_HR_ of the RT condition being explained by the IAT_HR_ achieved in the TM.

### IAT_bLa_

**Fig 4:**
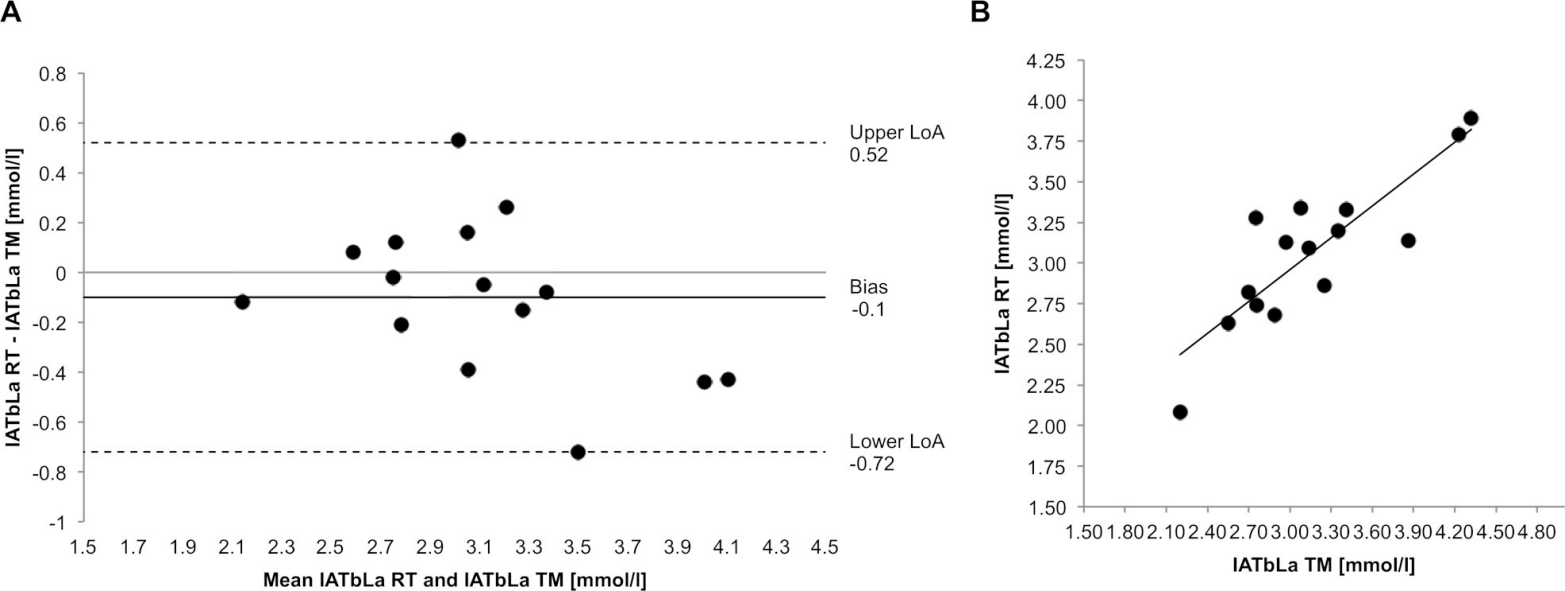
Agreement and strength of the relationship between TM and RT. (A) Bland-Altman plot to show the agreement of IAT_bLa_ and (B) Scatterplot of the IAT_bLa_ achieved for RT (y-axis) versus TM (x-axis) to show strength of relationship.

IAT_bLa_ was normally distributed (ΔRT *&* TM: p = 0.563). A two-tailed paired *t* test revealed no statistically significant difference between TM (3.16 ± 0.6, 95% CI 2.8–3.5 mmol/l) and RT (3.07 ± 0.46, 95% CI 2.8–3.3 mmol/l); p = 0.252; Fig 1C). The Bland-Altman plot (Fig 4A) showed a systematic bias of -0.1 mmol/l with a random error of 0.62 mmol/l (upper LoA: 0.52, lower LoA: -0.72 mmol/l). Correlation analysis (Fig 4B) shown a strong association (r = 0.856; p< 0.001) with 73.2% of the variability of IAT_bLa_ of the RT condition being explained by the IAT_bLa_ achieved in the TM.

### BLa_max_ and HR_max_

No significant differences in HR_max_ between TM (189 ± 8, 95% CI 185–193) and RT (188 ± 9, 95% CI 183–193) were found (p = 0.331). However, HR_max_ showed a strong correlation (r = 0.860, p< 0.001) with 74.0% of the variability in RT being explained by TM. Six participants achieved the same HR_max_ whereas five had a higher value in TM condition. Conversely, four recorded their highest heart rate in RT. The individual differences ranged from 1 to 11 bpm. HR_max_ was systematically lower for RT with a bias of 1.13 bpm and a random error of 8.54 bpm (upper LoA: 7.41, lower LoA: - 9.67 bpm). BLa_max_ between conditions was significantly different (TM: 10.38 ± 2.43, 95% CI 9.03–11.72; RT: 11.45 ± 2.75, 95% CI 9.92–12.97, p = 0.022) and this was strongly correlated (r = 0. 815, p< 0.001, R^2^ = 0.665). Overall, 12 out of 15 participants reached a higher bLa_max_ in RT. Individual differences ranged from 0.1–4.2 mmol/l. A bias and random error of 1.07 mmol/l and 3.14 mmol/l (upper LoA: 4.21, lower LoA: -2.07 mmol/l) were shown respectively.

### DIST

**Fig 5:**
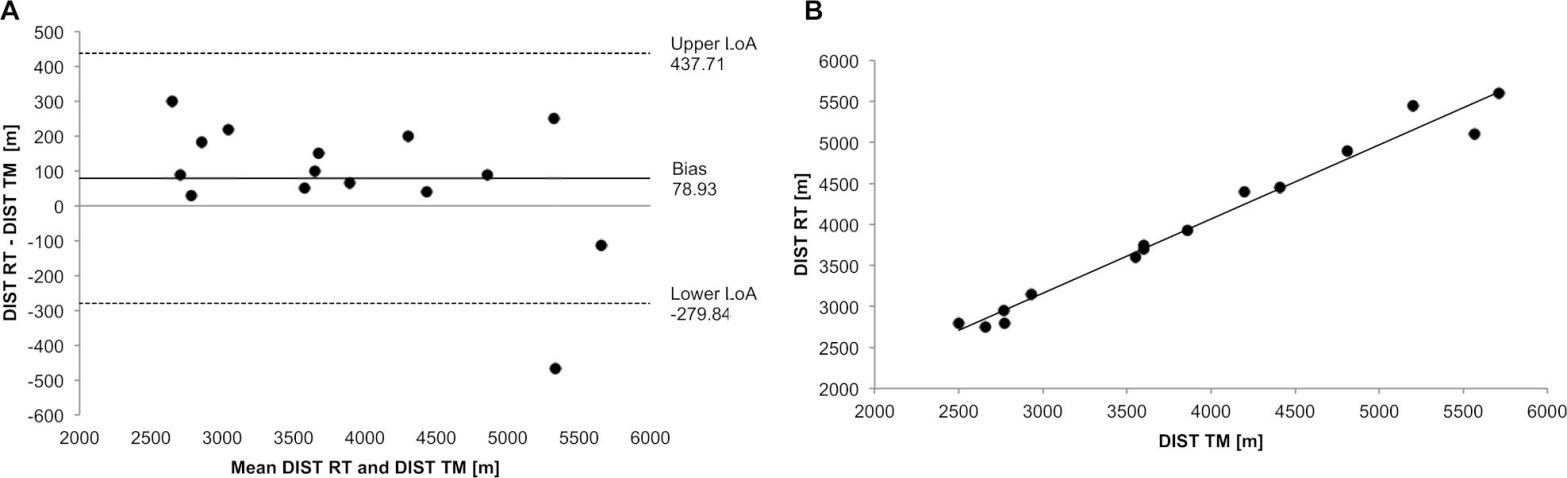
Agreement and strength of the relationship between TM and RT. (A) Bland-Altman plot to show the agreement of DIST and (B) Scatterplot of the DIST achieved for RT (y-axis) versus TM (x-axis) to show strength of relationship.

The assumption of normal distribution of the results for differences in DIST was not met (p = 0.006). The Wilcoxon signed rank test revealed a statistically significant difference between TM (3876 ± 1077, 95% CI 3280–4472 m) and RT (3955 ± 986 95% CI 3409–4501 m; p = 0.036). Only two participants covered fewer meters in RT, whereas 13 more in RT. The Bland-Altman plot (Fig 5A) showed a systematic bias of 78.93 m towards RT with a random error of 358.78 m (upper LoA: 437.71, lower LoA: -279.84 m). The correlation analysis (Fig 5B) revealed a strong positive association (r = 0.988; p< 0.001) with 97.6% of the variability in DIST in RT is explained by the DIST achieved in TM.

### RPE

Thirteen participants (87%) reported a maximal RPE value of 20/20 and the remaining two (13%) reported 19/20 after volitional termination of TM. In RT, 10 (67%), four (27%) and one participants (7%) expressed their level of exhaustion with 20/20, 19/20 and 18/20, respectively. Only five participant’s RPE readings deviated between both conditions. That is, four rated TM as more exhausting whereas one perceived higher exhaustion in RT. The RPE ratings at the IAT_speed_ were significantly different (TM: 15 ± 1, 95% CI 14–15; RT: 13 ± 1, 95% CI 13–14, p < 0.001) and had only a moderate correlation between conditions (r = 0.679, p < 0.005).

## Discussion

The aim of the study was to assess whether the inclination used on a motorized treadmill was sufficient to give valid training recommendations. The primary finding demonstrated that applying a 0.4% gradient is strongly correlated with the over-ground condition as expressed by IAT_speed_. Additionally, the distance covered by the runners was also highly correlated between conditions. To the knowledge of the authors’, this study represents the first to compare the IAT_speed_ in both a laboratory and outdoor setting using the same stepwise incremental protocol.

The results of the IAT measurements lay within the a priori defined LoA of ± 1 km/h. A systematic difference of 0.03 km/h suggests no practical implication for runner’s training management. The authors hypothesized that by using a 0.4% gradient on the treadmill the wind and ambient air resistance that occurs during OG could be accounted for. This could, in turn, enable clinicians to give credible outdoor training recommendations based on the runners’ treadmill performance. The results showed exceptional consistency when comparing IAT_speed_ of the two conditions. IAT_HR_ showed a more modest association (r = 0.746) and therefore, it could be argued that its prescriptive properties based on lab testing are not as reliable as that of IAT_speed._ Heart rate during exercise can be influenced by several factors that were not actively controlled in the current study, e.g. hydration status [25]. The results would indicate that, recommending the speed (km/h) at which a person should train is preferable over a target HR, as it is likely to reflect running at the anaerobic threshold.

In general, the use of lactate thresholds (e.g. IAT) is widely accepted as being an appropriate criterion when compared to other performance indicators e.g. VO_2max_ and HR. IAT_speed_ was chosen as the most important physiological parameter due to its strong prescriptive properties and reliability [8,26–28]. The IAT, as calculated according to Dickhuth (1999) [7], is one of approximately 25 proposed concepts to estimate MLSS [1] and has been previously used for performance diagnostics and control of training [26,29]. A strong correlation (range: r = 0.91–0.96) has been repeatedly observed when determining IAT_speed_ indirectly by comparing mean running speed on the track over 3 km to the MLSS obtained in the laboratory [30–32]. However, using IAT_speed_ obtained via direct bLa sampling in a field test has to be cross-validated using the same protocol in a laboratory-based setting. It can be reasonably speculated that if the 0.4% gradient on the treadmill over- or underestimated the conditions of OG running, then a shift in IAT_speed_ would have been seen. No such difference was reported in the present findings. Therefore, a 0.4% gradient can be considered accurate and IAT_speed_ as an important physiological marker when conducting validity studies and making valid training recommendations.

In a practical environment, it is very often the case that runners will opt for OG as their preferred modality of endurance training. This being the case, any information provided based on TM results must be valid and simulate real world conditions as well as having practical application. Many studies that employ the use of a motorized treadmill will often arbitrarily use a 1% gradient without any explanation [33–35]. Although unstated, it could be based on the findings of Jones (1996) [2], whom concluded that out of four different gradients (0%, 1%, 2% and 3%), running at a 1% gradient reflected most accurately OG conditions in terms of oxygen consumption at speed between 10.5 km/h and 18 km/h.

The 0.4% inclination used in the current study appeared to be a steep enough incline to simulate the conditions of an OG protocol that contained running speeds ranging from 6 to 22 km/h. To support this notion, not only were the calculated differences in IAT_speed_ and IAT_HR_ negligible, the overall performance as expressed by DIST was highly correlated between TM and RT. With the aforementioned factors considered, the accuracy of training recommendations are well supported by the present findings.

Perhaps the biggest limitation of the study may be attributed to the non-randomization of the design itself. This allows for potential motivation bias, whereby the participants try to beat their previous stage achieved on the TM. It should be said; however, that although a bias of 79 m in favor of OG was observed, there could be several other explanations to account for these systematic differences. It could be argued that runners perceive the same workload as easier running outside as suggested by the significantly lower RPE at IAT_speed_ in the RT condition (p< 0.001). However, the confidence intervals between conditions question the meaningfulness of that significant difference in RPE by 1 AU. The lower RPE could be due to runners being more comfortable and familiar with OG compared to unfamiliar settings of treadmill running, especially when running at maximum speed. In terms of practical application and training recommendations of the IAT_speed_, it operates under the assumption that the OG running will be constantly performed on a level terrain. In practice, an athlete would not be able to maintain their IAT_speed_ whilst running uphill.

The methodology could also be questioned as the termination criteria used for this study could have possibly resulted in a slight overestimation of DIST. The runners in the present study were afforded some leeway to reach the next 50 m mark whereas this would not have been practically possible for TM. Additionally, at high treadmill speeds, the fear of falling off or injury due to volitional fatigue could have culminated in premature termination of the test. A further potential cause of variability between conditions could have resulted from the lactate analyzer itself, as according to the manufacturer, the coefficient of variation is ≤1.5%. Finally, environmental and methodological factors cannot be controlled in RT. However, it has been reported that large temperature differences are not a significant factor when investigating the time to exhaustion and IAT_speed_ of endurance runners [36]. Therefore, it is likely that the temperature differences in the current study (TM: 25°C; RT: 20°C) would not confound the validity of the findings.

## Conclusion

The results reconfirm that prescriptions based on laboratory testing are accurate due to the high reliability of IAT_speed_ between the TM and RT conditions. Although not generalizable, the current study shows that revalidation of used protocols and facilities are mandatory to prescribe valid and up to date training recommendations to athletes and patients alike. This study offers a methodological approach whereby institutions can routinely revalidate their own treadmill-based recommendations.

## Supporting information

S1 Dataset Minimaldataset. (XLSX)
